# NRF2信号通路促进非小细胞肺癌增殖的研究进展

**DOI:** 10.3779/j.issn.1009-3419.2022.102.37

**Published:** 2022-10-20

**Authors:** 译萱 方, 心如 邹, 舒宁 胡, 俐俐 季

**Affiliations:** 226001 南通，南通大学医学院病理解剖学系 Department of Pathological Anatomy, School of Medicine, Nantong University, Nantong 226001, China

**Keywords:** 肺肿瘤, 增殖, NRF2, Lung neoplasms, Proliferation, NRF2

## Abstract

肺癌的发病率和死亡率位居全球癌症前列，非小细胞肺癌（non-small cell lung cancer, NSCLC）是肺癌的主要病理类型，且治疗手段有限，患者预后较差。细胞核因子E2相关因子2（nuclear factor E2-related factor, NRF2）信号通路在NSCLC中高度突变激活，并通过多种机制促进肺癌的恶性进展，以NRF2为靶点的治疗方案将为NSCLC患者提供新的治疗策略。本文将对NRF2通路的基本结构和反应途径、NRF2调节肺癌细胞增殖的机制，及NRF2抑制剂的研发进展进行综述。

## Abstract (en)

The morbidity and mortality of lung cancer ranks among the top cancers in the world. Non-small cell lung cancer (NSCLC) is the main pathological type of lung cancer, with limited treatment options and poor prognosis. The nuclear factor E2-related factor 2 (NRF2) signaling pathway is highly mutated and activated in NSCLC, and promotes the malignant progression of lung cancer through various mechanisms. NRF2-targeted therapy will provide new treatment strategies for patients with NSCLC. This article will review the basic structure and response pathways of the NRF2 pathway, the mechanism of NRF2 regulating lung cancer cell proliferation, and the research and development progress of NRF2 inhibitors.

肺癌的发病率和死亡率均位于全球癌症前列^[1]^，大约85%的肺癌患者被诊断为非小细胞肺癌（non-small cell lung cancer, NSCLC）^[2, 3]^。NSCLC起病隐匿，早期症状不明显，大部分患者在初诊时已经出现局部恶化或者转移症状，手术与放化疗作为治疗手段作用有限，预后较差，5年总体存活率低下^[4]^。因此，对NSCLC发病机制和治疗措施的研究迫在眉睫。

细胞核因子E2相关因子2（nuclear factor E2-related factor, NRF2）/Kelch样环氧氯丙烷相关蛋白1（Kelch-like ECH-associated protein 1, KEAP1）信号通路是体内应对过度氧化应激的重要防御通道，可以保护细胞不受应激伤害预防癌变，因此NRF2最初被认证为肿瘤抑制因子^[3]^。但癌症基因组图谱数据（The Cancer Genome Atlas, TCGA）及相关临床数据^[3, 5, 6]^显示NSCLC中常出现*NRF2*突变，尤其在肺鳞癌（lung squamous carcinoma, LUSC）中这一现象更频繁，超过1/3的LUSC患者发生NRF2通路异常活化。NRF2的生物活性调控机制以及它在肿瘤发展过程中的促癌作用一直是近年来肿瘤学研究的热点。研究^[7]^指出瘤细胞通过NRF2表达上调保护其在活性氧增高环境下存活而促进肿瘤增殖，并且有研究^[8]^发现NRF2是参与肿瘤代谢重编程的重要因子，可指挥瘤细胞利用葡萄糖和谷氨酰胺以加速肿瘤细胞增殖。针对NSCLC患者的研究^[9-11]^表明，NRF2通路持续激活可诱导肿瘤细胞的恶性增殖及对抗肿瘤药物产生耐药而导致治疗手段作用有限，使患者预后不良。以上证据表明NRF2可能在NSCLC（尤其是LUSC）的发生发展中起到促癌作用。鉴于NRF2在NSCLC发展过程中的重要作用，聚焦NRF2的治疗策略也逐渐受到重视，以NRF2为靶点的治疗方案将为NSCLC患者开发新型个体化抗癌疗法提供更全面、更深入的见解。

本文主要围绕NRF2/KEAP1信号通路的基本结构与反应途径以及NRF2促进NSCLC增殖的机制进行综述。此外，还将对目前NRF2抑制剂的研发进展进行阐述，旨在为以NRF2为靶点的NSCLC治疗方法提供新思路。

## 1 NRF2、KEAP1基本结构及经典反应途径

### 1.1 NRF2基本结构

NRF2于1994年被首次发现，是一种含碱性区域亮氨酸拉链（basic-region leucine zipper, bZIP）结构的抗氧化转录因子^[12, 13]^，包含7种NRF2-ECH同源结构域（NRF2-ECH homology, Neh），依次命名为Neh1-Neh7^[3]^。Neh1是调节NRF2活性的主要部位，可通过CNC-bZIP结构与小肌肉腱膜纤维肉瘤蛋白（small masculo-aponeurotic fibrosarcoma proteins, sMAF）组成二聚体，使NRF2可以识别并结合细胞核DNA中抗氧化反应元件（antioxidant response element, ARE）序列，继而启动下游靶基因转录活化；Neh2位于N端，该区域含有与KEAP1结合的DLG和ETGE两个高度保守序列；Neh3存在于C端，能与转录共激活因子CHD6结合，是转录激活的必要条件；Neh4和Neh5可协同Neh3共同促进NRF2活化；Neh6结构域中含有DSGIS和DSAPGS高度保守序列，与β-转导素重复蛋白（β-transducin repeat-containing protein, β-TrCP）识别并结合以促进NRF2的泛素化降解；Neh7与维甲酸X受体α（retinoic X receptor α, RXRα）相互作用，抑制NRF2靶基因转录^[3, 14-16]^。

### 1.2 KEAP1基本结构

KEAP1属于BTB-Kelch蛋白家族，在细胞质与Cullin3（CUL3）组成E3泛素连接酶复合体，随后与NRF2 Neh2部位结合降解NRF2^[3, 14, 15]^。KEAP1包含三个功能区域，分别为BTB、IVR和DGR区域^[14]^，每一个结构域对抑制NRF2活性具有重要作用。BTB区域参与结合CUL3泛素连接酶，使KEAP1发生同源二聚化^[3, 16]^；IVR区域与含有E3连接酶复合物和Roc1的CUL3蛋白相互作用，还具有核输出信号的共有序列，有利于KEAP1在细胞质中的定位，同时该区域存在丰富的半胱氨酸残基调节KEAP1活性^[16, 17]^；DGR又称为Kelch，该区域与NRF2 Neh2的DLG或ETGE序列结合，处于氧化应激时，DLG序列便从DGR区域中释放，阻断KEAP1对NRF2的降解^[3, 17, 18]^。

### 1.3 NRF2/KEAP1信号通路经典反应途径

当细胞处于非应激状态时，由于KEAP1 DGR区域与NRF2 Neh2结构域中的DLG/ETGE序列结合，促使KEAP1与CUL3形成E3泛素连接酶复合体，使NRF2在26S蛋白酶体中发生泛素化降解^[3, 15, 16, 19]^，此时NRF2处于低活性低表达状态。当细胞遭遇氧化应激刺激时，KEAP1结构中第151位氨基酸（Cys 151）受到共价修饰导致其功能失活，NRF2脱离KEAP1并转移到细胞核与下游多个基因启动子区域ARE序列结合^[14, 20]^，继而诱导抗氧化基因如依赖还原型辅酶Ⅰ/Ⅱ醌氧化还原酶[NAD(P)H: quinone oxidoreductase, NQO1]等表达增加^[15, 19]^，清除体内多余活性氧（reactive oxygen species, ROS），维持氧化还原平衡，使得细胞各项生物活动正常进行。

## 2 NRF2/KEAP1信号通路调节肺癌细胞增殖的机制

### 2.1 NRF2信号通路与PI3K/AKT/mTOR信号通路

磷脂酰肌醇-3激酶（phosphatidylinositol-3-kinase, PI3K）/AKT/哺乳动物雷帕霉素靶蛋白（mammalian target of rapamycin, mTOR）是调节细胞生长、代谢的主要通路^[21]^，常在肿瘤细胞中异常激活，导致肿瘤细胞恶性增殖，是引起肿瘤耐药的重要因素^[22]^。近几年在结直肠癌（colorectal carcinoma, CRC）、NSCLC等恶性肿瘤细胞以及一些正常细胞如神经元细胞、人食管上皮细胞和肾小球系膜细胞中均发现了NRF2通路与PI3K/AKT/mTOR通路的交联作用，表明两者的协同作用广泛存在于多种类型细胞中，并且两条通路共同作用使细胞更容易在应激环境下存活并增殖^[23-27]^。研究^[8]^发现，当PI3K/AKT/mTOR通路处于持续激活时，NRF2活性进一步增强，诱导细胞发生代谢重编程，促进A549细胞异常增殖。而在*NRF2*突变的LUSC细胞中可以发现PI3K/AKT/mTOR通路的异常活化，此时运用mTOR抑制剂可以显著抑制*NRF2*突变LUSC细胞增殖^[28]^，以上说明NRF2通路与mTOR通路相互影响共同推动肿瘤发展。RagD是mTOR通路激活的关键因子^[29, 30]^，已有研究^[28, 31]^表明NRF2可以通过调节NSCLC细胞中RagD转录活性激活mTOR通路促进细胞增殖。除上述机制外，PI3K/AKT/mTOR通路还可以通过其他方式调节NRF2信号转导。例如，零价铁纳米颗粒（Zero-valent-iron nanoparticle, ZVI-NP）可以通过激活NSCLC中腺苷酸活化蛋白激酶（AMP-activated protein kinase, AMPK）/mTOR通路，启动细胞内SCF/β-TrCP机制，增强对NRF2的降解作用，下调肿瘤的自我更新能力，抑制NSCLC细胞的恶性增殖^[32]^。此外，最近的一项研究^[33]^发现肺腺癌（lung adenocarcinoma, LUAD）细胞中存在一种新型NRF2靶基因即*CHML*/*Rep2*，该基因介导mTOR转位到溶酶体膜，在溶酶体膜上激活并启动下游靶基因磷酸化，从而驱动蛋白质翻译过程，而Rep2发生缺失则影响mTOR依赖的蛋白合成过程，降低细胞增殖。由此可见，NRF2与PI3K/AKT/mTOR信号通路的相互作用对NSCLC的恶性进展起到明显推动作用，可以为未来靶向两种通路的联合治疗提供新的建议。

### 2.2 NRF2信号通路与细胞周期因子

有文献^[34]^阐明细胞周期与肿瘤发展紧密相关，特定细胞周期因子的失调可能直接促进肿瘤细胞增殖。Malhotra等^[35]^利用染色质免疫共沉淀技术（chromatin immunoprecipitation, ChIP）对*NRF2*基因组进行分析后发现细胞周期调控抑制因子*Cdkn1a*和*Cdkn2b*是*NRF2*的靶基因。他们以NRF2^WT^、NRF2^-/-^和KEAP1^-/-^三种细胞为研究对象，诱导细胞*Cdkn1a*和*Cdkn2b*表达，结果显示KEAP1^-/-^细胞的增殖速率相比另外两种NRF2低表达或不表达的细胞显著提升^[35]^。细胞周期相关激酶20（cell cycle-related kinase 20, CDK20）是新发现的细胞周期调控因子，在肺癌细胞中CDK20可与NRF2竞争结合KEAP1促进癌细胞持续增殖，最终使得肿瘤细胞对抗肿瘤药物产生抵抗^[36]^。研究^[37]^发现NRF2是推动细胞周期从G_2_期向M期转变的重要调节因子，当NRF2功能受损时，细胞无法正常进入M期，有丝分裂过程被迫停止，细胞增殖中断。研究^[38]^显示miR-6077可以直接靶向CDKN1A和KEAP1保护NSCLC免受顺铂诱导的细胞周期阻滞和铁死亡影响，使细胞产生耐药现象，该结果进一步表明细胞周期可与NRF2通路协同调节NSCLC恶性发展。总体而言，NRF2与细胞周期间相互作用的探讨已经逐步成为热点，但NRF2与细胞周期相互调节的具体机制目前还处于起步阶段，未来还需要更多的研究去探讨深层机制。

### 2.3 NRF2信号通路与细胞代谢重编程

考虑到瘤细胞的高增殖率，肿瘤细胞通常会大量消耗微环境中的各种营养物质，因此癌细胞需重新规划其代谢，以确保在营养缺乏和压力的微环境下生存和增殖，这种现象我们通常称为细胞代谢重编程^[39, 40]^。近几年NRF2被认为是驱动肿瘤细胞代谢重编程的关键转录因子，NRF2通过上调葡萄糖-6-磷酸脱氢酶（glucose-6-phosphate dehydrogenase, G6PD）、磷酸葡萄糖脱氢酶（phosphogluconate dehydrogenase, PGD）等表达，增加细胞葡萄糖摄取，并引导细胞进入磷酸戊糖途径（pentose phosphate pathway, PPP）促进细胞增殖^[10]^。NRF2也可以通过调节从头合成核苷酸代谢途径相关酶如磷酸核糖焦磷酸酰胺转移酶（phosphoribosyl pyrophosphate amidotransferase, PPAT）、亚甲基四氢叶酸脱氢酶2（methylenetetrahydrofolate dehydrogenase 2, MTHFD2）为细胞生长和增殖提供必需的核苷酸原料^[10]^。有趣的是，NRF2在PI3K/AKT/mTOR通路持续激活或者*PTEN*基因缺失的条件下活性显著提高，可进一步增强代谢基因表达，促进肿瘤细胞增殖^[8]^，这一现象不仅表明NRF2参与细胞代谢过程重新分配肿瘤细胞所需营养，也再次证实了NRF2与PI3K/mTOR通路之间的交联作用。此外，Galan-Cobo等^[41]^发现NSCLC患者同时存在*KRAS*基因突变和丝氨酸/苏氨酸激酶STK11（serine/threonine kinase STK11, LKB1）缺失时，细胞在激活NRF2/KEAP1信号通路的同时启动谷氨酰胺代谢程序，增强NSCLC对谷氨酰胺抑制剂的敏感性。这些研究结果表明NSCLC中NRF2是参与肿瘤细胞代谢的重要信号转录因子。虽然细胞代谢是一个复杂的过程，目前对于氧化应激通路NRF2如何协同其他通路共同促进肿瘤细胞代谢增殖的机制研究尚未完全清楚，但是NRF2参与肿瘤细胞代谢增殖的作用为靶向治疗NSCLC患者提供了新的依据。

### 2.4 NRF2信号通路与铁死亡

铁死亡在肿瘤生长过程中所起的作用已经越来越受到大众的关注。铁死亡这一概念最早由Dixon于2012年提出^[42]^，是一种不同于细胞坏死、凋亡、自噬的新型细胞程序性死亡方式。细胞发生铁死亡时会出现细胞内游离铁增加、脂质过氧化物蓄积以及细胞线粒体形态变化三种主要的特征性改变^[42]^。溶质载体7家族成员11（solute carrier family 7 member 11, SLC7A11）和谷胱甘肽过氧化物酶4（glutathione peroxidase 4, GPX4）既是调控铁死亡的关键因子，也是NRF2的下游靶点^[43]^。研究^[44]^发现NSCLC中NRF2/KEAP1信号通路激活，SLC7A11表达随之上调，细胞内谷氨酸含量增多，使得肿瘤细胞微环境发生改变，进而诱导细胞对铁死亡抵抗性增加，最终导致细胞增殖失控、恶性进展加快。Gai等^[45]^发现NSCLC细胞可通过异位表达金属硫蛋白1D假基因（metallothionein 1D pseudogene, *MT1DP*）抑制NRF2活性而使细胞对铁死亡诱导剂Erastin诱导的铁死亡敏感，肿瘤细胞活性下降。然而也有部分NSCLC细胞对Erastin诱导的铁死亡不敏感并出现失控性增殖，此时联合运用Erastin和乙酰氨基酚（acetaminophen, APAP）可通过调节NRF2核易位，减少胞内谷胱甘肽含量，增加脂质过氧化物，抑制细胞活力，最终使此类细胞对铁死亡敏感性增强，抑制细胞过度增殖^[46]^。最近研究^[32]^发现ZVI-NP可以增强NRF2的泛素化降解过程，从而提升细胞内ROS水平使之发生过度氧化应激而诱导A549等肺癌细胞发生铁死亡，从而有效抑制肿瘤生长和转移。从上述研究结果来看，铁死亡作为一种新发现的细胞死亡形式，将打破肺癌治疗的瓶颈，为肿瘤治疗提供一种新策略。但是，NRF2通路参与铁死亡调控的机制还有待进一步研究，NRF2作为铁死亡相关基因的重要调节器，其在肺癌的恶性进展过程中具体发挥了何种作用还需要更多的研究去验证。

### 2.5 NRF2信号通路与线粒体功能

一系列证据^[47, 48]^表明NRF2/KEAP1信号通路与线粒体功能之间存在关联。线粒体是细胞重要的生命活动场所，为细胞的生长提供能量和营养物质，而线粒体功能紊乱会导致细胞坏死或凋亡，从而中断细胞增殖^[49]^。生理状态下，NRF2/KEAP1信号轴可与多种功能蛋白相互作用加强NRF2介导的线粒体功能，如PI3K、AKT、p62等可与NRF2竞争结合KEAP1，使NRF2不被降解并激活其介导的相关线粒体功能^[50]^。目前认为，*NRF2*过度表达引起的致癌作用部分源于线粒体功能加强和代谢重编程，线粒体功能增强使得肿瘤细胞增殖所需的营养得到充分补充，而当*NRF2*基因突变或缺失则会引起氧化应激损伤和线粒体代谢紊乱^[50]^。有研究^[47]^发现NRF2抑制剂鸦胆子苦醇（Brusatol, BRU）通过增强细胞内活性氧（reactive oxygen species, ROS）水平大量消耗谷胱甘肽（glutathione, GSH），破坏线粒体膜电位平衡，损害线粒体功能，使得肺癌细胞生长停滞在G_0_期/G_1_期，最终使细胞发生显著凋亡。目前，有关NRF2与线粒功能相互作用调节细胞生长的机制研究还鲜有报道，如果能够完全阐明两者之间的联系可能会给NRF2介导的细胞增殖调控机制带来新的认知。

## 3 NRF2抑制剂的研发

由于NRF2通路在NSCLC中普遍激活，并且研究^[51]^证明*NRF2*突变可促进癌细胞对药物产生抵抗而影响患者治疗，因此抑制肺癌细胞NRF2表达将会为缓解肺癌恶性进展提供新的视角。随着科技水平的提升，很多NRF2抑制剂也逐渐揭开“面纱”。我们目前所熟知的NRF2抑制剂主要从草本植物中提炼而来，包括黄酮类化合物、生物碱等^[51]^，这些抑制剂在提高肺癌细胞的药物敏感性方面效果显著。例如，Tang等^[52]^研究发现黄酮类化合物木樨草素（luteolin, LUT）通过急剧降低A549细胞中NRF2的mRNA和蛋白水平表达，导致NRF2无法与其下游靶基因ARE序列结合，并大量消耗细胞内谷胱甘肽，最终使A549等细胞增殖活力下降并对化疗药物敏感性增强。此外，将LUT与顺铂联合使用会对肿瘤细胞生长产生更明显的抑制效用^[52]^。然而LUT并未在动物实验和人体临床实验中进行更深入的研究，因而想要真正了解LUT对人体是否有其他副作用以及能否投入临床使用还需要更多的实验来检验。BRU是2011年被确认的一种NRF2抑制剂，该药物从鸭胆子属植物中提取纯化^[53]^。研究^[54]^发现BRU可以通过损伤A549细胞DNA以及促进ROS生成从而提升A549对放化疗药物敏感性。Ren等^[53]^进一步发现BRU与顺铂等化疗药物联合使用明显诱导细胞发生凋亡，相比单独使用化疗药物，BRU的加入显著抑制了肺肿瘤的生长。研究^[55]^发现BRU可直接靶向Skp1抑制NSCLC细胞生长和侵袭，是治疗NSCLC的潜力药物。然而有报道分析^[53, 56]^指出BRU具有抑制广谱蛋白翻译的特性，能对大多数蛋白质尤其是短半衰期蛋白均起到降低表达的作用，这种无差别抑制作用使得BRU无法作为NRF2的特异性靶向药物使用。虽然天然NRF2抑制剂在众多细胞实验中取得了显著进展，但在与人体环境更为相似的体内实验中还处于缓慢发展阶段，很多天然抑制剂能否被用于临床还需要更多研究才能证明。随着科研技术的发展，一些新型合成的NRF2抑制剂逐步进入大众视野。Singh研究团队^[57]^通过对临床化合物的高通量筛选，发现ML385能与转录因子NRF2的DNA结合域特异性结合，阻断其与下游靶基因启动子区相互作用而抑制靶基因的转录与表达，并且研究发现相对于NRF2野生型，*NRF2*突变型肺癌细胞在ML385刺激后表现出明显的NRF2通路抑制效应^[57]^，同时ML385在与卡铂等化疗药物联合使用后抗肿瘤效果更显著，ML385可进一步增强肺癌细胞对化疗药物的敏感性^[57, 58]^。因此ML385被预测将作为NRF2特异性抑制剂成为NSCLC尤其是*NRF2*突变肺癌患者的新靶向药。然而作为一种有治疗前景的新型药物，还需要更多的体内实验及后续临床实验来验证其作为抗肿瘤药物的有效性和安全性，同时ML385其抗肿瘤的具体分子机制还有待于更进一步的研究。

## 4 结论与展望

NRF2可以联合多种机制参与并协调肿瘤细胞的生长过程，是肿瘤生长与增殖的重要调控因素。近年来随着医疗技术的发展，许多增殖相关的基因被证实受到NRF2的转录调控，但由于NRF2信号通路的复杂与多变，迄今为止尚不能完全解读其调控细胞增殖的具体分子机制，仍然需要进一步探索。NRF2抑制剂的研发为NSCLC的治疗提供了新的方向，但由于这类药物缺乏可靠的临床证据，未来能否运用到肺癌患者的治疗中还需要更多的数据佐证。令人期待的是，靶向*NRF2*突变的药物研发，将会是一种有潜力的NSCLC特异性治疗方案，未来需要专门针对*NRF2*突变NSCLC患者开展临床研究以实现更优化的精准治疗。
